# Preparation of Iron-Loaded Granular Activated Carbon Catalyst and Its Application in Tetracycline Antibiotic Removal from Aqueous Solution

**DOI:** 10.3390/ijerph16132270

**Published:** 2019-06-27

**Authors:** Ling Pan, Yanzhi Cao, Ji Zang, Qinqing Huang, Lin Wang, Yingsheng Zhang, Shisuo Fan, Jun Tang, Zhengxin Xie

**Affiliations:** 1 School of Resources and Environment, Anhui Agricultural University, Hefei 230036, China; 2 Key Laboratory for Agri-Food Safety, Anhui Agricultural University, Hefei 230036, China

**Keywords:** granular activated carbon, tetracycline antibiotics, Fenton-like reaction, heterogeneous catalyst

## Abstract

The removal of tetracycline antibiotics from water is currently an important environmental issue. Here we prepared an iron-loaded granular activated carbon catalyst (GAC-Fe) through a one-step calcination method to remove tetracycline antibiotics from aqueous solution. The GAC-Fe was characterized by Fourier transform infrared absorption spectroscopy, X-ray photoelectron spectroscopy, and X-ray diffraction analysis. The effect of different influencing factors on the removal behavior of tetracycline antibiotics was studied, such as the solid-to-liquid ratio, H_2_O_2_ dosage, environmental temperature, initial pH, and contact time. The removal mechanism was explored through Fe ion dissolution and a free radical quenching experiment. The results show that the optimum solid-to-liquid ratio was 3.0 g∙L^−1^ and the suitable H_2_O_2_ dosage was 1.0 mL (3%). The applicable environmental temperature was 25 °C and the appropriate pH value was 2.0. The removal rate of tetracycline antibiotics tended to be stable in a contact time of 600 min. The main mechanism of tetracycline antibiotic removal by GAC-Fe was heterogeneous catalytic reaction through iron ion leaching and free radical inhibition experiment. The hydroxyl radical played a major role during the removal process. The partially dissolved iron ions initiated a homogeneous catalytic reaction. However, heterogeneous catalytic degradation was the main reaction. The GAC-Fe could still remove tetracycline antibiotics after five cycles, especially for methacycline and minocycline. Our work suggests that the GAC-Fe catalyst has potential as a remediation agent for tetracycline antibiotics in aqueous solution.

## 1. Introduction

Tetracycline antibiotics are among the most used antibiotic group for human and animal health [1,2]. Due to their low cost and high antimicrobial activity, tetracycline antibiotics are particularly marketable in developing countries [1]. In China, the annual usage of tetracycline and oxytetracycline was approximately 1450 and 1360 tons during 2013, respectively [3]. After medication, more than 70% of tetracycline antibiotics are excreted into the environment via the urine and feces of humans and animals [1]. In addition, tetracycline antibiotics cannot be completely removed by wastewater treatment plants [4]. As a result, tetracycline antibiotics have been widely detected in aquatic environments [2,5,6,7]. Tetracycline antibiotics can cause adverse effects on different species of aquatic organisms [8,9]. Perhaps most importantly, the residue of tetracycline antibiotics in aquatic environments can disrupt the structure and function of microbial communities, and induce the emergence of antibiotic resistance among bacteria, posing a great threat to human health and disease treatment [10,11]. Therefore, the removal of tetracycline antibiotics from water is crucial.

The treatment methods for antibiotic pollutants in wastewater mainly include adsorption, chemical precipitation, oxidation–reduction, ion exchange, and the biological method [12]. Through comprehensive consideration of the removal effect, low cost, simple operation, safety, and economy, an advanced oxidation method has been increasingly used for the removal of antibiotics in wastewater [13,14].

Fenton advanced oxidation reaction is widely studied and is of interest, but there are some problems in the homogeneous Fenton method, such as the high chromaticity of the degraded wastewater, the reaction occurrence at a lower pH, the production of iron sludge, and the difficulty of separating Fe^2+^ from wastewater [15]. Thus, it is considered that Fe^2+^ can be fixed on an appropriate carrier to allow it to participate in the degradation reaction in the form of a heterogeneous catalyst. A heterogeneous Fenton reaction effectively overcomes the above problems to some extent. The carrier for the heterogeneous catalyst includes sludge [16], carbon nanotubes [17], zeolite [18], silica [18], alumina [19], clay minerals [20], iron oxides [21], and so forth [22].

Among these carriers, carbon-based heterogeneous catalysts such as porous carbon, carbon nanotubes, and activated carbon (AC) have been increasingly investigated [23]. Because of the wide source, large specific surface area, and developed pore size, in addition to its good adsorption effect, carbon-based material can be an ideal choice for iron-loaded heterogeneous catalysts. Moreover, iron oxide loaded on the AC can further improve catalytic activity [24,25,26].

Iron-loaded carbon-based catalysts have been used to remove contaminants from water bodies [27,28,29,30,31]. Duarte et al. [32] studied the preparation of a heterogeneous Fe/AC Fenton-like catalyst and used the catalyst for the removal of the azo-dye Orange II. Nguyen et al. [33] prepared magnetically loaded AC for the removal of methyl orange dye from aqueous solution. Zhang et al. [34] used ACloaded with zero-valent iron as a heterogeneous catalyst in an electro-Fenton process for the removal of methyl orange from aqueous solution. The above studies showed that an iron-loaded carbon-based catalyst had a better removal effect than a pollutant in aqueous solution.

Carbon-based Fenton catalysts that can be used to degrade antibiotics in wastewater have gradually appeared. Ma et al. [35] synthesized heterogeneous carbon nanotubes/FeS Fenton-like catalyst and used it for the removal of antibiotics from aqueous solution. Wan et al. [36] studied the removal of sulfamethazine antibiotics using cerium–iron–graphene nanocomposite as catalyst by a Fenton-like process. Jaafarzadeh et al. [37] prepared a powder AC/Fe_3_O_4_ hybrid composite as a highly efficient heterogeneous catalyst for Fenton oxidation of tetracycline. Shi et al. [38] investigated the heterogeneous Photo-Fenton degradation of norfloxacin with Fe_3_O_4_ multi walled carbon nanotubes in aqueous solution. However, the preparation of heterogeneous catalysts on granular activated carbon (GAC) through loading iron ions and the simultaneous degradation of multiple tetracycline antibiotics have rarely been reported.

In this study, an Fe ion loaded granular activated carbon (GAC-Fe) catalyst was prepared by one-step calcination with ferrous ions supported by GAC as a carrier, and the effects of solid–liquid ratio, H_2_O_2_ dosage, ambient temperature, initial pH, and contact time on antibiotic removal were investigated. The removal mechanism was revealed by Fe ion dissolution and a free radical quenching experiment. This research can provide a theoretical basis and technical reference for the resource utilization of GAC and the control of antibiotic wastewater pollution.

## 2. Materials and Methods

### 2.1. Materials

GAC was purchased from Hefei Huamo Environmental Protection Technology Co. Ltd. Other reagents, including 98% H_2_SO_4_, NaOH, H_2_O_2_, and FeSO_4_, were purchased from Sinopharm chemical reagent Co. Ltd. Six kinds of tetracycline antibiotics (tetracycline, oxytetracycline, aureomycin, doxycycline, methacycline, and minocycline) were purchased from Shanghai Yuanye Reagent Company, China. The physicochemical properties of target tetracycline antibiotics are presented in the Supporting Information (Table S1).

### 2.2. Preparation of Iron-Loaded Granular Activated Carbon Catalyst

GAC (7 g) was added to a beaker containing 100 mL FeSO_4_ solution (0.2 mol∙L^−1^, pH = 4.10). The beaker was then stirred at 25 °C for 120 min on a magnetic stirrer. After it was dried in the oven, the iron-loaded granular activated carbon was put in a muffle furnace for calcination at 400 °C and kept for 90 min. After the iron-loaded granular activated carbon was cooled to room temperature, it was taken out from the furnace and washed with distilled water until the eluent was close to neutral. Furthermore, the solid was put into an oven at 105 °C for 1 h drying. The obtained catalyst was named GAC-Fe.

### 2.3. Antibiotic Removal Experiment

(1) Adsorption of tetracycline antibiotics by GAC and GAC-Fe: in order to determine the adsorption effect of the GAC and GAC-Fe catalysts on antibiotics, 100 mL of antibiotic mixture solution with a concentration of 10 mg·L^−1^ for each antibiotic was added to a 250 mL conical flask. The mixture solution contained six kinds of tetracyclines, including tetracycline, oxytetracycline, aureomycin, doxycycline, methacycline, and minocycline. The pH value was adjusted to 2.0 with hydrochloric acid, and then 3 g·L^−1^ of GAC was added to the flask. The conical flask was placed on a constant-temperature oscillator. The temperature was controlled at 35 °C, and the speed of oscillator was set at 135 rpm for 1 h. The concentration of antibiotics in the filtrate was determined, and the removal rate of the sample was calculated.

(2) Effect of H_2_O_2_: in order to determine the effect of H_2_O_2_ on antibiotic removal, 100 mL of antibiotic mixture solution with a concentration of 10 mg·L^−1^ for each antibiotic was added to a 250 mL conical flask. The pH value was adjusted to 2.0 with hydrochloric acid, and then 1.0 mL/100 mL of H_2_O_2_ (3%) was added to the flask. Other procedures and conditions were the same as the description in the adsorption of tetracycline antibiotics by GAC and GAC-Fe.

(3) Effect of different influencing factors: antibiotic mixture solution (100 mL) with a concentration of 10 mg·L^−1^ for each antibiotic was added to a 250 mL conical flask. The pH value was adjusted to a specific value with hydrochloric acid. A certain amount of GAC-Fe catalyst was then added, and a certain amount of H_2_O_2_ was added to initiate the degradation experiment. Different influencing factors on antibiotic removal were investigated. The set amounts of GAC-Fe were 0.2, 0.5, 1, 2, 3, and 4 g·L^−1^. The H_2_O_2_ dosage was set at 0.1, 0.5, 1.0, 1.5, 2.0, and 3.0 mL/100 mL, respectively. The environmental temperature was controlled at 25, 35, 45, and 55 °C. The pH value was adjusted to 2.0, 3.0, 4.0, 5.0, 6.0, and 7.0. When the effect of one influencing factor on antibiotic removal was investigated, all other influencing factors in the experiment were set as follows: GAC-Fe amount, 0.3 g·L^−1^; H_2_O_2_ dosage, 1.0 mL/100 mL; temperature, 25 °C; pH, 2.0.

(4) Effect of contact time: to study the effect of contact time on antibiotic removal by GAC-Fe, the interval times for sample collection were set as 5, 10, 20, 30, 60, 120, 240, 360, 480, and 600 min. The concentration of antibiotics in the filtrate was determined, and the removal rate of the sample was calculated. Other procedures and conditions were the same as the adsorption experiment.

(5) Iron leaching experiment: the experimental procedure was the same as the effect of the contact time experiment. A sample was taken for filtration, and the concentration of iron ions in the filtrate was determined by an atomic absorption spectrophotometer (ZEEnit 700P, Analytik Jena, Jena, Germany).

(6) Effect of tertiary butanol on antibiotic removal: antibiotic solution (100 mL) with a concentration of 10 mg·L^−1^ was added to a 250 mL conical flask. The pH value was adjusted to 2.0 with hydrochloric acid, and 3 g·L^−1^ of GAC was added in the flask, and then 1.0 mL/100 mL of H_2_O_2_ (3%) was added to the flask. Tertiary butanol was then added to the solution. The volume ratios for tertiary butanol and antibiotic solution were 1:100 and 1:500, respectively. The conical flask was placed on a constant-temperature oscillator. The temperature was controlled at 35 °C, and the speed of the oscillator was set at 135 rpm for 1 h. The concentration of antibiotics in the filtrate was determined, and the removal rate of the sample was calculated.

### 2.4. Recycling Experiment

A certain volume of 100 mL antibiotic solution with a concentration of 10 mg·L^−1^ was added to a 250 mL conical flask. The pH value was adjusted to 2.0 with hydrochloric acid, and then 1.0 mL of H_2_O_2_ (3%) was added to the flask. The conical flask was placed on a constant-temperature oscillator. The temperature was controlled at 35 °C, and the speed of oscillator was set at 135 rpm for 1 h. The concentration of antibiotics in the filtrate was determined, and the removal rate of the sample was calculated. The solid and liquid were then separated by centrifugation. The solid catalyst was cleaned with methanol, and then washed with pure water, and last dried in vacuum. Five cycles were conducted.

### 2.5. Characterization of Catalyst

The mineral composition and type of GAC-Fe was characterized by X-ray diffraction (XRD; Bruker, D8 Advance, Karlsruhe, Germany). The functional groups of GAC-Fe before and after the reaction were measured by Fourier transform infrared (FTIR) spectroscopy (ThermoFisher Scientific Co., Waltham, MA, USA). Changes in surface elements, morphology, and relative distribution before and after the GAC-Fe reaction were analyzed by X-ray photoelectron spectroscopy (XPS; Thermo-VG Scientific, Escalab250, Waltham, MA, USA).

### 2.6. Measurement of Tetracycline Antibiotics

Tetracycline antibiotics were measured by high-performance liquid chromatography (Waters, Millford, SC, USA) with an ultraviolet detector. A BEH-C18 column (2.1 × 1000 mm, 1.7 μm) was applied for all chromatographic separations. The mobile phase comprised 0.1% (v/v) formic acid (A) and acetonitrile (B) using a gradient program of 90% A in 0–14 min, 75% A in 14–16 min, 100% B in 16–17.10 min, 90% A in 17.10–20 min. The flow rate was maintained at 0.2 mL·min^−1^, and the column temperature was held at 40 °C. The detection wavelength at 254 nm was selected to acquire chromatograms. The injection volume was 10 μL.

## 3. Results and Discussion

### 3.1. Characterization of GAC-Fe

**Figure 1:**
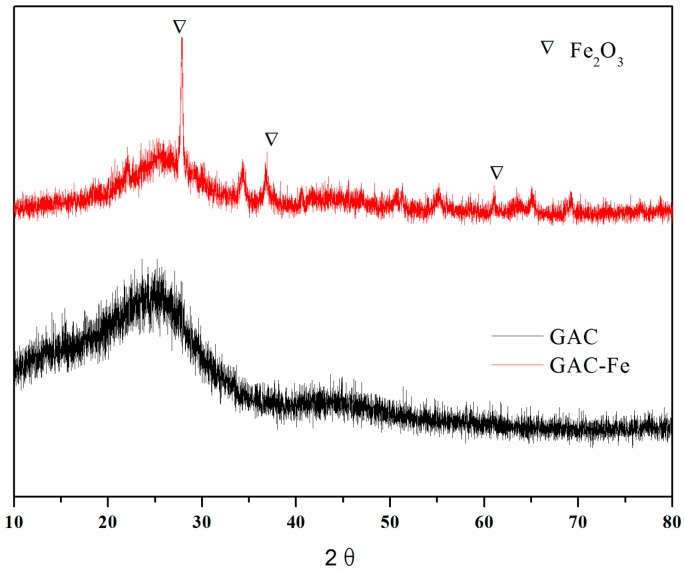
X-ray diffraction (XRD) spectrum of granular activated carbon (GAC) and granular activated carbon catalyst (GAC-Fe).

Figure 1 shows the XRD spectrum of GAC and GAC-Fe. As shown in the spectrum, the peak shape of GAC in the range of 20–30 (2θ) is not sharp, indicating that amorphous carbon dominated in the GAC. Fe_2_O_3_ could be detected in the GAC-Fe because the iron ion could be oxidized to Fe_2_O_3_ under air calcination. Fe_2_O_3_ could be formed through calcination under air atmosphere [39,40].

**Figure 2:**
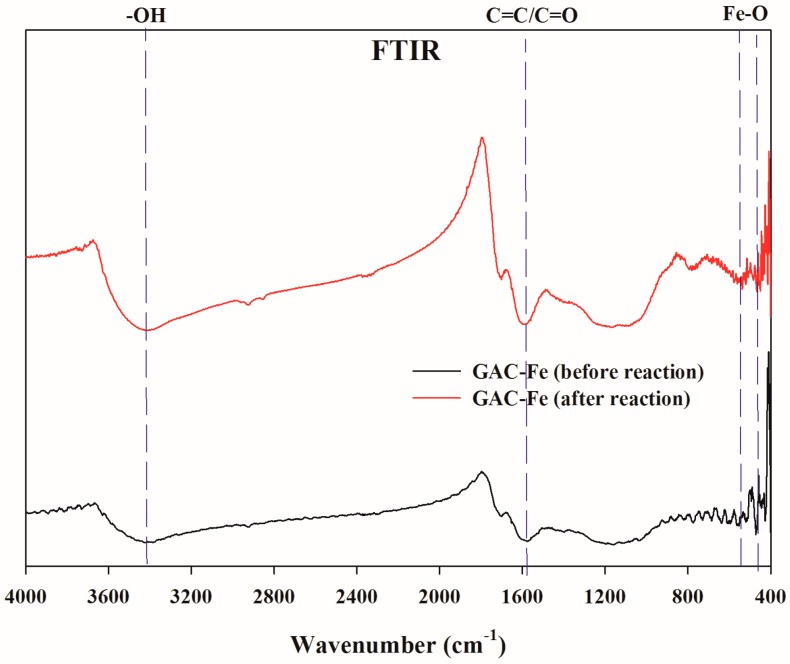
Fourier transform infrared (FTIR) spectrum of GAC and GAC-Fe.

The FTIR spectra of GAC-Fe before and after the reaction are displayed in Figure 2. The main functional groups in the GAC-Fe included −OH (3423 cm^−1^), C=C/C=O (1577 cm^−1^), and Fe–O (472 cm^−1^) [27,28,29]. After the GAC-Fe reaction with tetracycline antibiotics, these functional groups could also be detected, especially for Fe–O group, indicating that the catalyst had the potential to regenerate. It should be noted that all peaks were not strong in the present study. This phenomenon could be due to the decomposition of functional groups in the precursor under high temperature pyrolysis.

**Figure 3:**
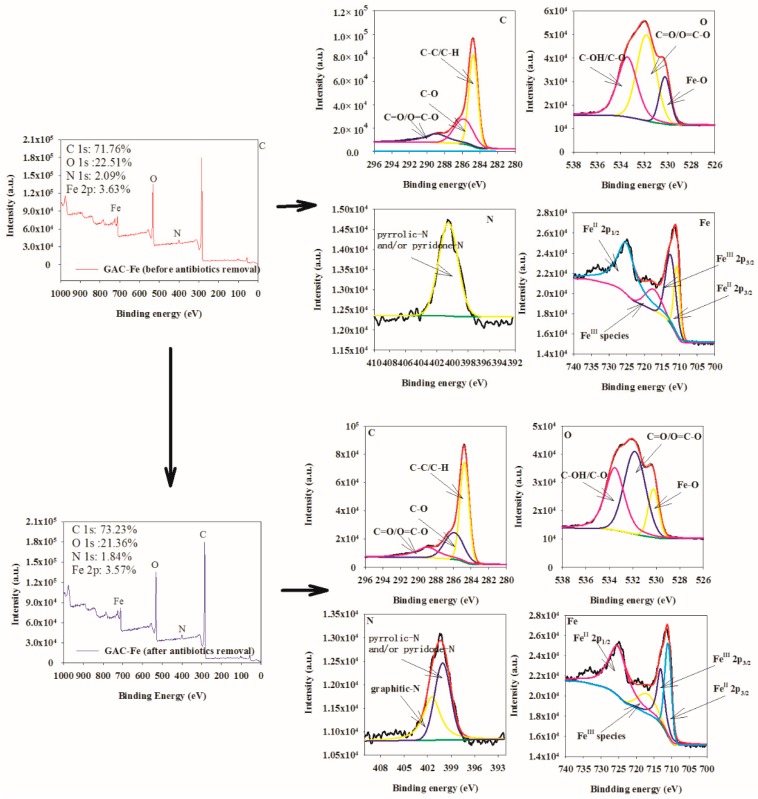
X-ray photoelectron spectroscopy (XPS) of GAC-Fe before and after antibiotic removal.

Figure 3 shows the XPS spectrum of GAC-Fe before and after the removal of antibiotics. Both GAC and GAC-Fe could detect the existence of C, N, O, and Fe. The relative content of Fe decreased from 3.63% to 3.57% after the reaction. The Fe in the catalyst after the reaction indicated that the GAC-Fe had potential for recycling.

Functional groups of different elements can be obtained by peak-splitting fitting. The fitted C 1s XPS spectrum (Figure 3) displays three peaks at 284.75, 285.85, and 288.93 eV, which are attributed to C–C/C–H, C–O, and O=C–O/C=O, respectively [41]. The fitted O 1 s XPS spectrum (Figure 3) presented three peaks at 530.24, 531.81, and 533.44 eV attributed to Fe–O, C=O/O–C=O, and O=C–O/C–OH, respectively [42]. The N 1s XPS spectrum was located at 400.53 eV, corresponding to pyrrolic N and/or pyridine N [43]. The peaks at the binding energies of 709.0–710.0 eV, 710.0–712.0 eV, and 724.0–725.0 eV were ascribed to Fe^II^ 2p_3/2_, Fe^III^ 2p_3/2_, Fe^II^ 2p_1/2_, respectively [44,45,46]. A satellite peak at around 718.0 eV confirmed the presence of Fe^III^ species on the surface of the samples. According to the XPS analysis before and after action, the position and area of peak of O–, N– and Fe–containing groups changed. Therefore, the XPS analysis revealed that Fe had been successfully loaded on GAC and that Fe–, O– and N–containing groups may be involved in the removal process of tetracycline antibiotics.

### 3.2. Removal of Tetracycline Antibiotics under Different Systems

**Figure 4:**
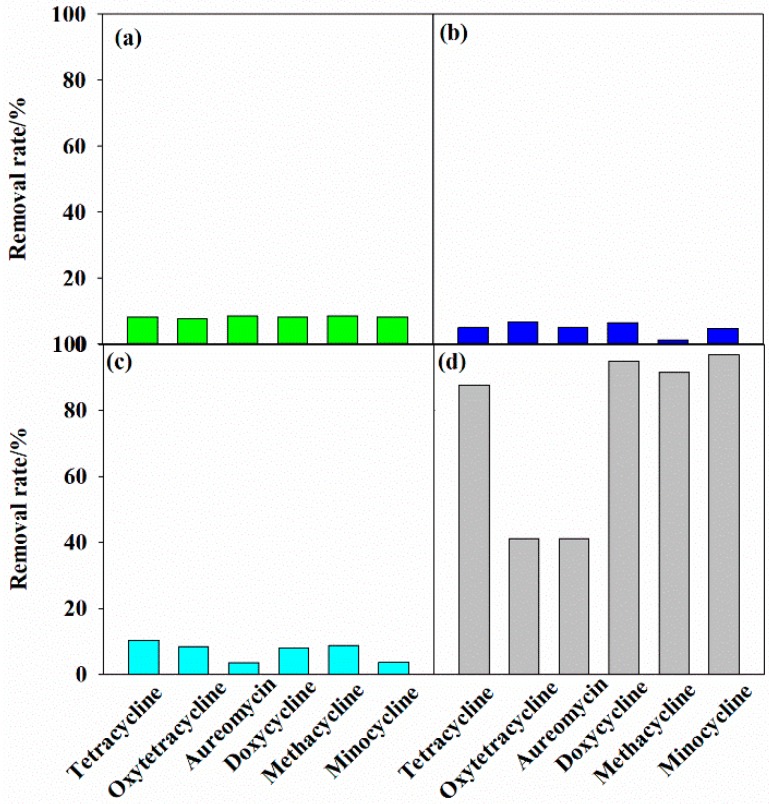
Removal rate of tetracycline antibiotics under different systems: (**a**) Antibiotics adsorption on GAC; (**b**) Antibiotics adsorption on GAC-Fe; (**c**) Antibiotic removal by H_2_O_2_ without GAC-Fe (**d**) Antibiotic removal by GAC-Fe (0.3 L^−1^) and H_2_O_2_ (1 mL/100 mL).

The removal of tetracycline antibiotics under GAC adsorption, GAC-Fe adsorption, H_2_O_2_ degradation, and GAC-Fe/H_2_O_2_ system was investigated. The adsorption effect of tetracycline antibiotics on GAC is shown in Figure 4a. The removal rates of tetracycline, oxytetracycline, aureomycin, doxycycline, methacycline, and minocycline were 8.27%, 7.75%, 8.52%, 8.19%, 8.58%, and 8.25%, respectively. Thus, the removal rate of tetracycline antibiotics on GAC was limited.

The adsorption effect of tetracycline antibiotics on GAC-Fe is shown in Figure 4b. The removal rates of tetracycline, oxytetracycline, aureomycin, doxycycline, methacycline, and minocycline were 4.94%, 6.65%, 5.10%, 6.49%, 1.19%, and 4.87%, respectively. Hence, GAC-Fe was also limited to remove tetracycline antibiotics. The poor antibiotic removal by GAC and GAC-Fe may be attributed to the limited surface area, pore structure and functional groups in two materials. In addition, the removal rate of GAC-Fe was lower than that of GAC. A possible explanation for this observation was that the iron oxide occupied the adsorption site on GAC-Fe.

The removal rate of tetracycline antibiotics by H_2_O_2_ (without adding GAC-Fe) is presented on Figure 4c. H_2_O_2_ could remove antibiotics to some extent, and the removal rate was less than 10%. H_2_O_2_ is a weak oxidant that can produce ·OH, which degraded the tetracycline antibiotics.

The removal rate of tetracycline antibiotics by GAC-Fe under H_2_O_2_ catalysis is shown in Figure 4d. The removal effect of tetracycline antibiotics by GAC-Fe under H_2_O_2_ was greatly improved. The removal rates of tetracycline, oxytetracycline, aureomycin, doxycycline, methacycline, and minocycline were 87.63%, 41.13%, 41.09%, 94.95%, 91.44%, and 96.83%, respectively. Thus, the GAC-Fe-catalyzed H_2_O_2_ system had excellent removal effects for doxycycline, methacycline, and minocycline, good removal effects on tetracycline, and certain removal effects on oxytetracycline and aureomycin. The increase in removal efficiency may be due to the fact that GAC-Fe catalyzed H_2_O_2_ to produce more ·OH. The relatively low removal rate of oxytetracycline an aureomycin compared to other antibiotics may be related to the limited active site on the GAC-Fe, the physicochemical properties such as molecular weight and structure, or the selective degradation of GAC-Fe. Further research is needed to explore the exact mechanisms for different remove efficiencies of tetracycline antibiotics.

### 3.3. The Removal Rate of Antibiotics under Different Influencing Factors

#### 3.3.1. Catalyst Dosage

**Figure 5:**
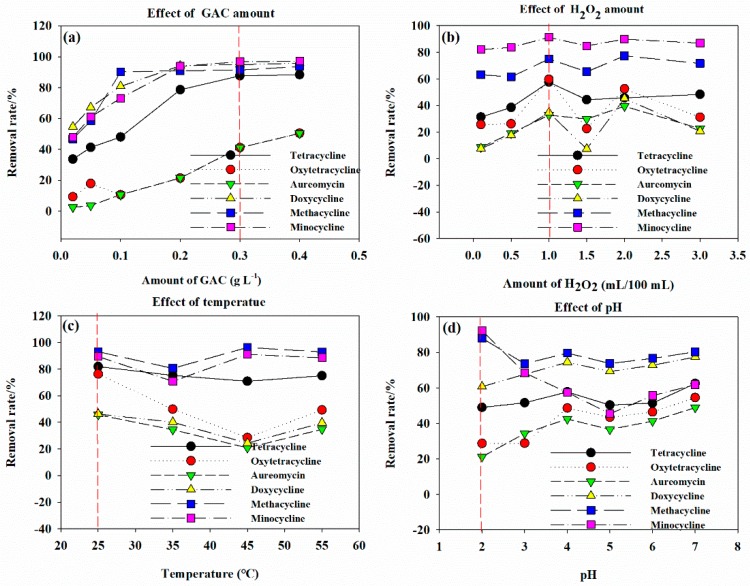
Effects of GAC amount (**a**), H_2_O_2_ amount (**b**), temperature (**c**) and pH (**d**) on the removal of tetracycline antibiotics by GAC-Fe.

The effect of GAC-Fe dosage on the removal rate of antibiotics is shown in Figure 5a. With the increase in catalyst dosage, the removal rate of antibiotics by GAC-Fe also increased. GAC-Fe had a poor removal effect on oxytetracycline and aureomycinas compared with the other four antibiotics. The removal rate of methacycline and minocycline by GAC-Fe under the catalyst H_2_O_2_ was larger than 90%. The removal rate of tetracycline by GAC-Fe under the catalyst H_2_O_2_ was larger than 87%. The removal rate of oxytetracycline and aureomycin by GAC-Fe under the catalyst H_2_O_2_ was only larger than 40%. Considering the removal effect and economy, the optimum catalyst dosage was 0.3 g (3.0 g∙L^−1^ solid-to-liquid ratio).

#### 3.3.2. H_2_O_2_ Dosage

The influence of H_2_O_2_ dosage on tetracycline antibiotic removal is shown in Figure 5b. With the increase in H_2_O_2_ dosage, the removal rate of tetracycline antibiotics by GAC-Fe was stable. Compared with the other four tetracycline antibiotics, GAC-Fe had a better removal effect for methacycline (>60%) and minocycline (>80%). The removal rate of other four antibiotics was less than 60%. After 60 min of reaction, when the H_2_O_2_ dosages were 1.0 and 2.0 mL, the removal rates of methacycline were 75.12% and 77.41%, respectively, and the removal rates of minocycline were 90.28% and 89.85%, respectively. Considering the actual effect, we finally selected 1.0 mL as the dosage of H_2_O_2_.

#### 3.3.3. Environmental Temperature

The influence of environmental temperature on catalysis reaction has two aspects. On the one hand, increasing the environmental temperature can improve the activity of the catalyst and speed up the reaction. On the other hand, increasing the environmental temperature can reduce the adsorption effect of GAC-Fe. The influence of environmental temperature on tetracycline antibiotic removal is shown in Figure 5c. When the temperature increased from 25 to 55 °C, the removal rate of terramycin obviously decreased, those for tetracycline, aureomycin, doxycycline slightly decreased, and the removal rate of methacycline, minocycline remained almost unchanged. When the environmental temperature was 25 °C, the removal rates of tetracycline, oxytetracycline, aureomycin, doxycycline, methacycline, and minocycline were 81.94%, 76.31%, 45.68%, 46.33%, 93.05%, and 89.47%, respectively. When the environmental temperature was 55 °C, the removal rates of tetracycline, oxytetracycline, aureomycin, doxycycline, methacycline, and minocycline were 75.05%, 49.31%, 34.76%, 39.65%, 92.87%, and 88.62%, respectively. The different removal behavior of the six antibiotics may be related to the difference in the physicochemical properties of six tetracycline antibiotics such as pKa, molecular weight, solubility and log*K*_ow_. Our results suggested that raising the temperature was not beneficial for the removal of tetracycline antibiotics by GAC-Fe. Thus, 25 °C was chosen as the experimental temperature because of the mild and easily controlled condition.

#### 3.3.4. Initial of pH Solution

The influence of initial pH on tetracycline removal is shown in Figure 5d. With the increase in initial pH, the removal rates of tetracycline, oxytetracycline, aureomycin, and doxycycline by GAC-Fe increased. However, the removal rate of methacycline and minocycline by GAC-Fe decreased with the increase in initial pH. The appropriate pH value for tetracycline, oxytetracycline, aureomycin, and doxycycline removal was 7.0, and the suitable pH value for methacycline and minocycline removal was 2.0. Therefore, the pH value was adjusted to 2.0 in the subsequent experiment. That the optimal removal rate of different antibiotics happened at significantly different pH was likely related to the molecular structure of tetracyclines. As shown in Table S1, the six antibiotics have different functional groups in structure, resulting in their different acid dissociation constants (pKa). Consequently, the speciation of these antibiotics could be different at the same pH. It has been previously reported the speciation of tetracycline had significant effects on its removal by graphene oxide [47]. Thus, our results suggested that the pH was an important factor influencing the degradation process of tetracycline antibiotics by GAC-Fe.

### 3.4. Effect of Contact Time on Tetracycline Antibiotic Removal by GAC-Fe

**Figure 6:**
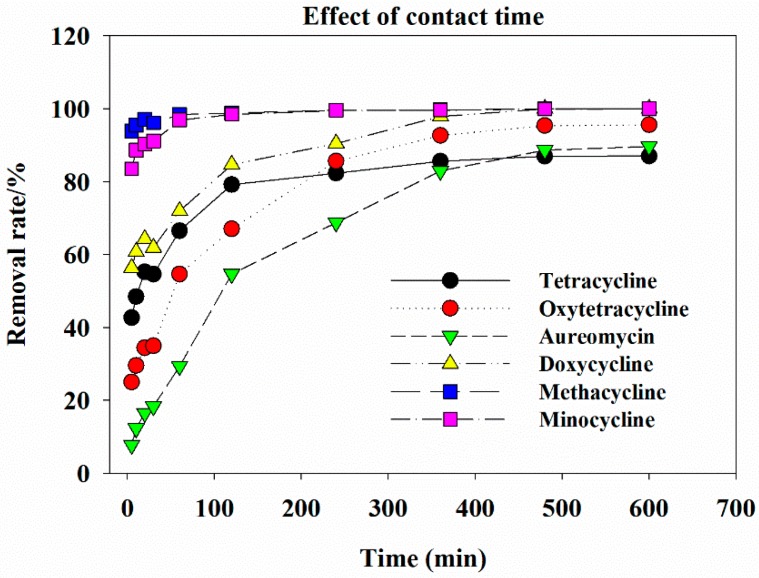
Effect of contact time on antibiotics removal.

The effect of contact time on tetracycline antibiotic removal by GAC-Fe is displayed in Figure 6. With the increase in contact time, the removal of tetracycline antibiotics by GAC-Fe first increased and then tended to be stable.

After 5 min of reaction, the removal rates of tetracycline, oxytetracycline, aureomycin, doxycycline, methacycline, and minocycline were 42.69%, 24.96%, 7.72%, 56.31%, 93.96%, and 83.51%, respectively. After 60 min of reaction, the removal rates of tetracycline, oxytetracycline, aureomycin, doxycycline, methacycline, and minocycline were 66.53%, 54.63%, 29.33%, 71.97%, 98.42%, and 96.92%, respectively. After 600 min of reaction, the removal rates of tetracycline, oxytetracycline, aureomycin, doxycycline, methacycline, and minocycline were 87.01%, 95.61%, 89.67%, 100%, 100%, 100%, respectively. Thus, longer contact time is beneficial for the removal of tetracycline antibiotics. The removal of methacycline, minocycline was quicker than that for the other four antibiotics. The removal rate tended to stabilize when the contact time was >600 min.

### 3.5. The Mechanism of Tetracycline Removal by GAC-Fe

#### 3.5.1. Iron Ion Leaching and Homogeneous Reaction

**Figure 7:**
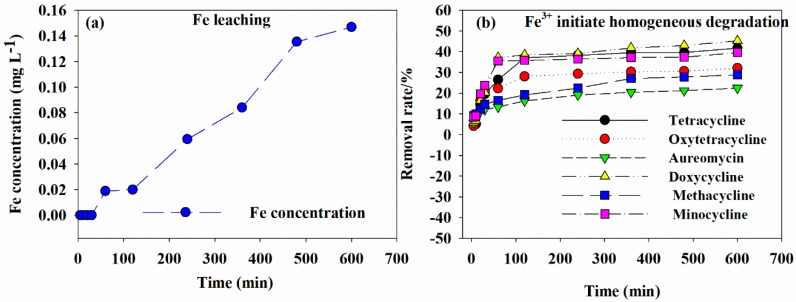
Fe leaching (**a**) and homogeneous reaction of Fe^2+^ (**b**).

The iron ion leaching during the antibiotic removal process by GAC-Fe is shown in Figure 7a. The iron ions could be detected after 60 min. The concentration of iron ions increased with the increase in contact time. The maximum concentration of iron ion reached 0.15 mg·L^−1^. As seen from Figure 7a,b, the iron ion leaching was aligned with the removal rate of antibiotics. Additionally, the removal of antibiotics was related to the degradation process. Thus, the dissolution of iron ions can easily induce homogeneous catalytic reaction and lead to the reduction of active sites in the catalyst.

In order to investigate the influence of homogeneous reaction, an experiment was carried out to study the impact of iron ions (0.15 mg·L^−1^, Fe^3+^) on tetracycline removal without addition of GAC-Fe catalyst. The result is presented in Figure 7b. After 5 min of reaction, the removal rates of tetracycline, oxytetracycline, aureomycin, doxycycline, methacycline, and minocycline were 4.3%, 4.22%, 5.72%, 5.87%, 9.13%, and 8.47%, respectively. After 60 min of reaction, the removal rates of tetracycline, oxytetracycline, aureomycin, doxycycline, methacycline, and minocycline were 26.49%, 22.29%, 13.37%, 37.26%, 16.58%, and 35.50%, respectively. After 600 min of reaction, the removal rates of tetracycline, oxytetracycline, aureomycin, doxycycline, methacycline, and minocycline were 41.77%, 32.10%, 22.50%, 45.30%, 28.89%, and 39.56%, respectively. Hence, it could be speculated that iron ions leaching from the GAC-Fe catalyst could induce the homogeneous reaction. Comparison of the result with the effect of contact time suggested that the heterogeneous catalytic reaction might play a dominant role during the tetracycline antibiotic degradation by GAC-Fe.

#### 3.5.2. Inhibition of Free Radical Reaction

In order to investigate the removal mechanism of tetracycline antibiotics by GAC-Fe, free radical trapping agent (tertiary butyl alcohol) was added to the reaction system to explore the reaction type [23,24]. The volume ratios of tertiary butyl alcohol and reaction solution were set at 1:100 and 1:500.

**Figure 8:**
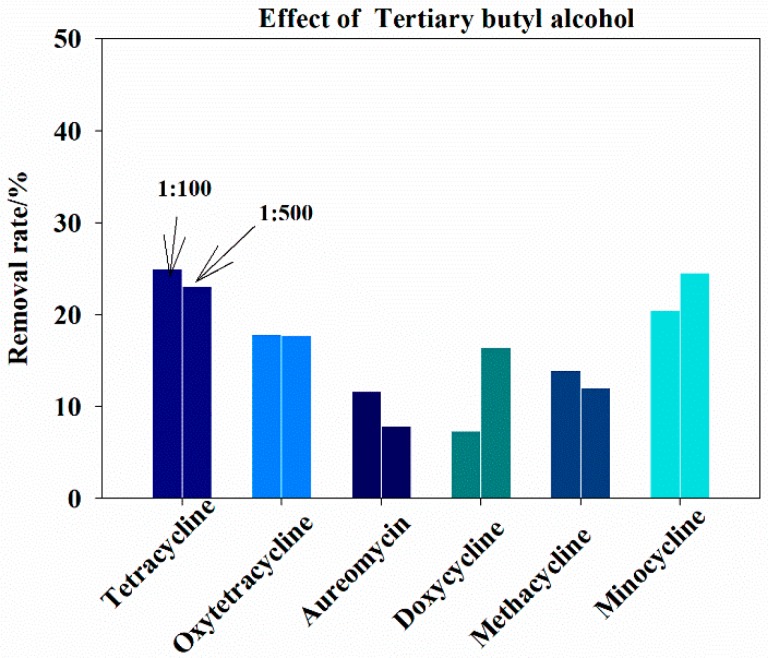
Effects of tert-butanol on tetracycline removal.

The inhibition of free radical reaction is shown in Figure 8. When the volume ratio was 1:100, the removal rates of tetracycline, oxytetracycline, aureomycin, doxycycline, methacycline, and minocycline by GAC-Fe were 24.95%, 17.0%, 11.60%, 7.33%, 13.96%, and 20.45%, respectively. When the volume ratio was 1:500, the removal rates of tetracycline, oxytetracycline, aureomycin, doxycycline, methacycline, and minocycline by GAC-Fe were 23.10%, 17.73%, 7.88%, 16.43%, 12.00%, and 24.53%, respectively. Thus, it can be seen that the main reaction mechanism for tetracycline antibiotics degradation by GAC-Fe was free radical reaction. The type of free radical was a hydroxyl radical (·OH), and the main mechanism was heterogeneous catalytic degradation. Interestingly, the removal rate of doxycycline and minocycline increased at higher tert-butanol dosage. Compared to the physicochemical properties of six tetracycline antibiotics, doxycycline and minocycline had higher log*K*_ow_. Thus, higher tert-butanol dosage may influence the distribution of doxycycline and minocycline, and further enhance the degradation effect of doxycycline and minocycline.

#### 3.5.3. Tetracycline Antibiotic Removal Mechanism by GAC-Fe

The degradation mechanism of antibiotics by a heterogeneous catalyst in the Fenton reaction has been reported in previous studies [48,49,50,51]. Overall, the main steps for tetracycline antibiotics by GAC-Fe with the catalysis of H_2_O_2_ were as follows. First, the tetracycline antibiotics were adsorbed on GAC-Fe. Subsequently, as indicated by the changes in the position and area of Fe^II^ 2p_3/2_ and Fe^III^ 2p_3/2_, the heterogeneous catalysis reaction occurred on the surface of GAC-Fe. Meanwhile, H_2_O_2_ could also partly degrade tetracycline antibiotics. Furthermore, the dissolution of iron ion from the catalyst could induce the homogeneous catalysis reaction to better remove the antibiotics. Lastly, the removal rate tended to stabilize when the H_2_O_2_ was completely consumed. However, the removal rate of six different tetracycline antibiotics by GAC-Fe antibiotics was different, and might be related to the chemical structure, functional groups, and species of antibiotics.

### 3.6. Effect of GAC-Fe Catalyst Recycling

**Figure 9:**
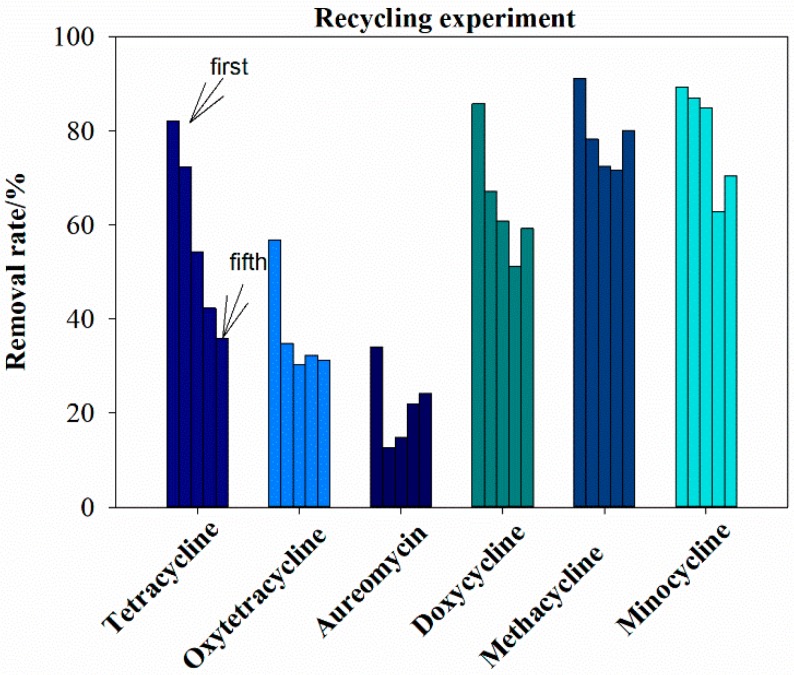
Recycling effect on tetracycline removal.

The recycling effect is an important index to assess the performance of a catalyst. As shown in Figure 9, with the increase in cycles, the removal rate of antibiotics by GAC-Fe presented a decreasing trend. When we used the catalyst for the first time, the removal rates of tetracycline, oxytetracycline, aureomycin, doxycycline, methacycline, and minocycline were 82.13%, 56.92%, 34.05%, 85.75%, 91.11%, and 89.35%, respectively. When we applied the GAC-Fe catalyst for the fifth time, the removal rates of tetracycline, oxytetracycline, aureomycin, doxycycline, methacycline, and minocycline were 35.93%, 31.20%, 24.18%, 59.25%, 80.11%, and 70.40%, respectively. Here, the removal rate for tetracycline, oxytetracycline, aureomycin, and doxycycline was less than 50%. However, the removal rates for methacycline and minocycline were more than 50%. After five recycling repetitions of reaction, the GAC-Fe catalyst could also partly degrade the tetracycline antibiotics. Hence, the GAC-Fe had potential for practical application. The reasons for the decline in antibiotic removal by GAC-Fe involved the mass loss of catalysts, dissolution of the iron ion, activation sites being covered, and pore blockage.

## 4. Conclusions

GAC was used as a carrier, and iron ion was loaded through the calcination method to prepare a GAC-Fe Fenton-like catalyst. The optimum solid-to-liquid ratio was 3.0 g·L^−1^, and the suitable H_2_O_2_ dosage was 1.0 mL (3%). The applicable environmental temperature was 25 °C, and the appropriate pH value was 2.0. The removal rate tended to stabilize after 10 h. The GAC-Fe catalyst still had a removal rate for tetracycline antibiotics after five recycling repetitions, especially for methacycline and minocycline. The iron leaching and free radical quenching experiment showed that the heterogeneous catalysis reaction was the main mechanism. The hydroxyl radical played a major role during the degradation process. Partially dissolved iron ions could induce a homogeneous catalytic reaction, but the heterogeneous catalytic dominated.

## Supplementary Materials

The following are available online at https://www.mdpi.com/1660-4601/16/13/2270/s1, Table S1: Physicochemical properties of target tetracycline antibiotics.
