# Supplementary material for: Preparation of Iron-Loaded Granular Activated Carbon Catalyst and Its Application in Tetracycline Antibiotic Removal from Aqueous Solution
Source: Int J Environ Res Public Health. 2019 Jun 27;16(13):2270. doi: 10.3390/ijerph16132270 (PMC6651779; doi:10.3390/ijerph16132270)
Supplement: Supplementary file 1 [file ijerph-16-02270-s001.pdf]

Table S1. Physicochemical properties of target tetracycline antibiotics.

Compound	CAS No.	M.W.	Formula	Solubility (in water, mg/L)	logK_{ow}	pK_a	Structure
Tetracycline	60-54-8	444.4	$C_{22}H_{24}N_2O_8$	231	−1.3	3.32<br>7.78<br>9.58	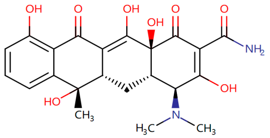
Oxytetracycline	79-57-2	460.4	$C_{22}H_{24}N_2O_8$	313	−0.9	3.22<br>7.46<br>8.94	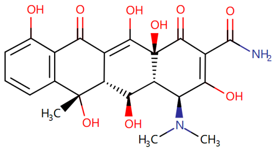
Aureomycin	57-62-5	478.88	$C_{22}H_{23}ClN_2O_8$	630	−0.62	3.58<br>7.97	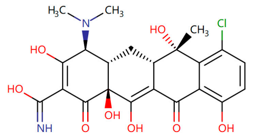
Doxycycline	564-25-0	444.44	$C_{22}H_{24}N_2O_8$	630	−0.02	3.02<br>7.97<br>9.15	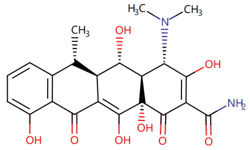
Methacycline	914-00-1	442.42	$C_{22}H_{22}N_2O_8$	7550	−1.37	2.88<br>7.44	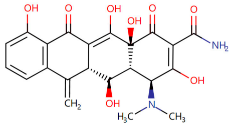
Minocycline	10118-90-8	457.48	$C_{23}H_{27}N_3O_7$	52,000	0.05	2.8<br>5.0<br>7.8<br>9.3	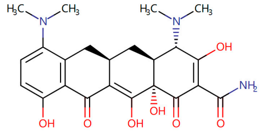
